# Ezrin Regulates Ca^2+^ Ionophore-Induced Plasma Membrane Translocation of Aquaporin-5

**DOI:** 10.3390/ijms222413505

**Published:** 2021-12-16

**Authors:** Shin-ichi Muroi, Yoichiro Isohama

**Affiliations:** Laboratory of Applied Pharmacology, Faculty of Pharmaceutical Sciences, Tokyo University of Science, 2641 Yamazaki, Noda 278-8510, Japan; shin36000903@gmail.com

**Keywords:** aquaporin-5, ezrin, trafficking, membrane water permeability, exocrine gland

## Abstract

Aquaporin-5 (AQP5) is selectively expressed in the apical membrane of exocrine glands, such as salivary, sweat, and submucosal airway glands, and plays important roles in maintaining their secretory functions. Because AQP5 is not regulated by gating, localization on the plasma membrane is important for its water-permeable function. Ezrin is an ezrin–radixin–moesin family protein that serves as a crosslinker between the plasma membrane and actin cytoskeleton network. It plays important roles in translocation of various membrane proteins to mediate vesicle trafficking to the plasma membrane. In this study, we examined the effects of ezrin inhibition on membrane trafficking of AQP5. Ezrin inhibition selectively suppressed an ionomycin-induced increase in AQP5 translocation to the plasma membrane of mouse lung epithelial cells (MLE-12) without affecting the steady-state level of plasma membrane AQP5. Taken together, our data suggest that AQP5 translocates to the plasma membrane through at least two pathways and that ezrin is selectively involved in a stimulation-dependent pathway.

## 1. Introduction

Aquaporins (AQPs) are water-selective channel proteins that allow rapid movement of water across the plasma membrane in secretory and adsorptive cells. Among the 13 isoforms of AQPs identified to date, AQP5 is selectively expressed in the apical membrane of secretory glands, such as salivary, sweat, and airway submucosal glands [1,2], and alveolar epithelial cells. Numerous studies have indicated that AQP5 plays critical roles in maintaining normal exocrine functions and that changes in the amount of cell surface AQP may contribute to abnormal water secretion in various diseases such as Sjögren’s syndrome bronchitis and cystic fibrosis. The water channel function of AQP5 is not regulated by structural gating. Furthermore, several lines of evidence have indicated that the amount of AQP5 is regulated by both gene expression and translocation between the plasma membrane and intracellular space. Translocation of AQP5 is affected by several receptor and signaling systems. Cyclic AMP and a protein kinase A (PKA)-dependent pathway have been shown to increase translocation from the intracellular space to the plasma membrane of bronchial epithelial, salivary gland, and sweat gland cells. The importance of phosphorylation at Ser156 in loop D has been suggested for such regulation [3]. Lipopolysaccharides also increase AQP5 translocation to the plasma membrane, which is mediated by a p38 MAP kinase-dependent mechanism [4]. This mechanism has been shown to be involved in other AQPs [5,6]. Additionally, several reports have shown that an increase in intracellular Ca^2+^ following activation of M3 acetylcholine receptor or TRPV4 as well as treatment with a Ca^2+^ ionophore stimulates translocation of AQP5 from the intracellular space to the plasma membrane of salivary gland cells [7,8,9]. A TRPV4-dependent mechanism has been shown in AQP4 translocalization [10]. Importantly, these regulatory mechanism for translocalization of AQPs did not affect total protein levels.

Ezrin–moesin-radixin (ERM) proteins serve as crosslinkers between the plasma membrane and actin cytoskeleton network. Ezrin is the most studied ERM protein. Ezrin and other ERM proteins have a C-terminal FERM domain, which interacts with the cytoplasmic domain of membrane and scaffolding proteins, such as Na^+^/H^+^ exchange regulatory factors, and an N terminal actin-binding domain that binds to F-actin [11,12]. These proteins play important roles in membrane protein and vesicle trafficking [13]. For example, ERM proteins regulate trafficking of H^+^-K^+^-ATPase [14], cystic fibrosis transmembrane conductance regulator [15], and other membrane transporters. Moreover, it has been suggested that ERM proteins regulate trafficking of AQP1 [16], AQP2 [17,18], and AQP3 [19]. In the present study, we investigated the effects of ezrin inhibition on translocation of AQP5 from the intracellular space to plasma membrane as well as membrane water permeability in mouse lung epithelial cells.

## 2. Results

### 2.1. Stimulation-Dependent Membrane Trafficking of AQP5 Does Not Occur in Ezrin-DN-Transfected Cells

**Figure 1:**
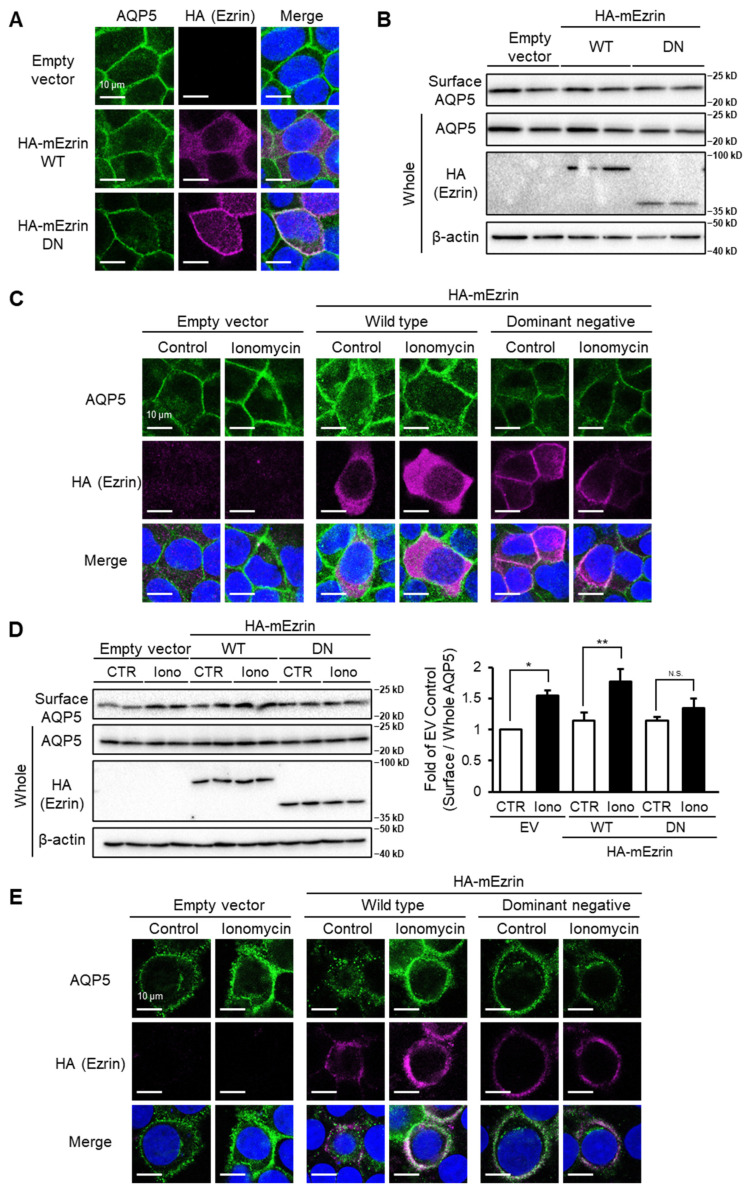
Ezrin-DN inhibits stimulation-dependent plasma membrane translocation of AQP5. MLE-12 cells were transfected with wildtype or dominant negative (lacking actin-binding domain) ezrin. Subcellular localization of AQP5 was analyzed by immunofluorescence (**A**), and the amount of cell surface AQP5 was assessed by Western blotting (**B**). Cells were treated with ionomycin (1 µM) for 15 min, after which cellular localization of AQP5 was analyzed by immunofluorescence (**C**) and the amount of cell surface AQP5 was assessed by cell surface biotinylation and Western blotting (**D**). HSG cells were transfected with wildtype or dominant negative ezrin, after which cells were treated with ionomycin (1 µM) for 15 min. Subcellular localization of AQP5 was analyzed by immunofluorescence (**E**). Blue staining in merge image indicates DAPI (nuclei). Data represent the mean ± S.E. (*n* = 3–6). * *p* < 0.05 and ** *p* < 0.01.

We first examined whether ezrin is required for steady-state localization of AQP5 in the plasma membrane. Expression plasmids for wildtype ezrin (ezrin-WT) or dominant negative ezrin (ezrin-DN) that lacked the C-terminal actin-binding domain were prepared and transfected into MLE-12 cells. There were no differences in subcellular localization of AQP5 between ezrin-WT- and ezrin-DN-transfected cells (Figure 1A). We also determined the amount of cell surface AQP5 by surface biotinylation and Western blotting. The amount of cell surface AQP5 in ezrin-DN-transfected cells was unchanged compared with that in ezrin-WT-transfected cells (Figure 1B), which suggests that ezrin is not involved in steady-state localization of AQP5 in the plasma membrane. We next investigated the effect of ezrin-DN transfection on Ca^2+^ ionophore ionomycin-induced translocation of AQP5 from the intracellular space to plasma membrane. In empty vector- or ezrin-WT-transfected cells, ionomycin treatment changed the subcellular localization of AQP5 from the intracellular space to plasma membrane (Figure 1C), which suggests that this reagent stimulates plasma membrane trafficking of AQP5. Interestingly, ionomycin did not change the subcellular localization of AQP5 in ezrin-DN transfected cells. Additionally, ionomycin did not change the amount of cell surface AQP5 in ezrin-DN-transfected cells, but it was significantly increased in empty vector- or ezrin-WT-transfected cells (Figure 1D). Consistently, in salivary gland cell line HSG, ionomycin increased plasma membrane-associated AQP5 in empty vector- or ezrin-WT-transfected cells, but not in ezrin-DN-transfected cells (Figure 1E). These results suggest that ezrin is required for stimulation-dependent plasma membrane trafficking of AQP5 in lung epithelial cells and exocrine cells.

### 2.2. Ezrin Inhibition Suppresses Stimulation-Dependent Membrane Trafficking of AQP5

**Figure 2:**
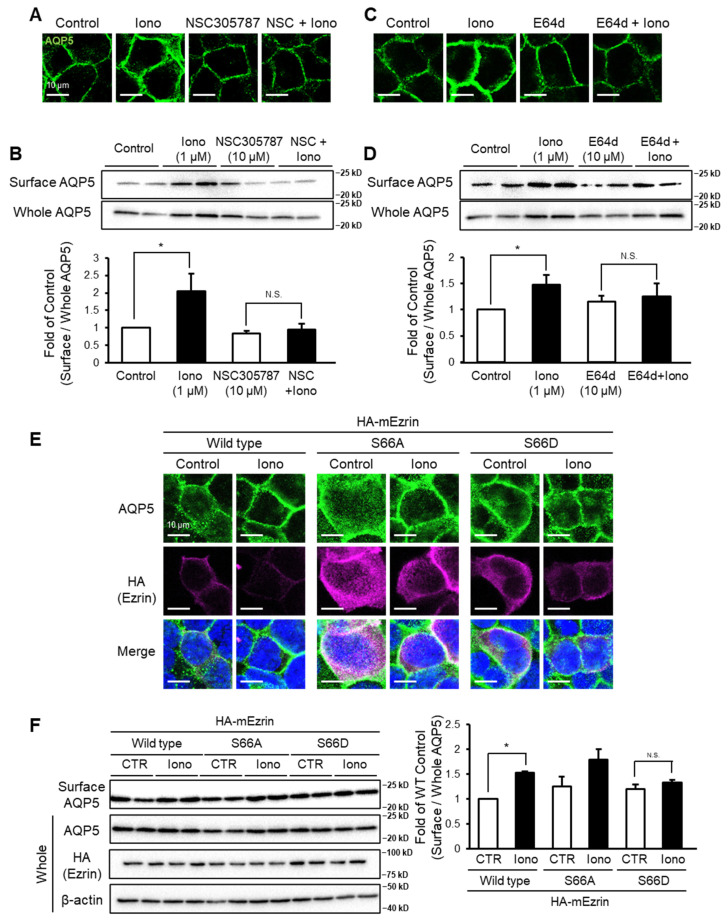
Ezrin inhibition decreases stimulation-dependent membrane trafficking. MLE-12 cells were treated with ionomycin (1 µM) and NSC305787 (10 µM) for 15 min. Subcellular localization of AQP5 was analyzed by immunofluorescence (**A**). The amount of cell surface AQP5 was assessed by cell surface biotinylation and Western blotting (**B**). MLE-12 cells were pretreated with E64d (10 µM) for 30 min and then treated with ionomycin (1 µM) for 15 min, after which subcellular localization of AQP5 (**C**) and the amount of cell surface AQP5 (**D**) were determined. MLE-12 cells were transfected with wildtype, S66A, or S66D (uncleavable mutant) ezrin and then stimulated with ionomycin (1 µM) for 15 min, after which subcellular localization of AQP5 (**E**) and the amount of cell surface AQP5 (**F**) were assessed. Data represent the mean ± S.E. (*n* = 3–4). * *p* < 0.05.

To confirm the ezrin requirement for stimulation-dependent plasma membrane trafficking of AQP5, we examined the effect of NSC305787 that directly binds to the ezrin FERM domain and inhibits its function [20]. NSC305787 (10 µM) suppressed ionomycin-induced translocation of AQP5 to the plasma membrane (Figure 2A). The increase in the amount of cell surface AQP5 induced by ionomycin was also inhibited by cotreatment with NSC305787 (Figure 2B). Ezrin is cleaved by calpain, which is essential for ezrin functions [21,22]. We therefore determined whether calpain inhibition suppressed stimulation-dependent membrane trafficking of AQP5 in MLE-12 cells. Pretreatment with E64d (10 µM), a cysteine protease inhibitor, suppressed AQP5 translocation to the plasma membrane induced by ionomycin (Figure 2C). The increase in the amount of cell surface AQP5 induced by ionomycin was also attenuated by E64d (Figure 2D). In cells transfected with an ezrin mutant (S66D) resistant to calpain cleavage, subcellular localization of AQP5 was not changed by ionomycin (Figure 2E). AQP5 localization was changed by ionomycin in ezrin-WT or ezrin mutant with alanine substitution for phosphorylation site of PKA (S66A) transfected cells. Furthermore, the amount of AQP5 in the cell surface fraction did not change after ionomycin treatment of ezrin-S66D transfected cells, whereas ionomycin increased the amount of cell surface AQP5 in ezrin-WT or -S66A-transfected cells (Figure 2F).

### 2.3. Ezrin-Mediated Stimulation-Dependent Membrane Trafficking of AQP5 Is Associated with an Increase in Membrane Water Permeability

**Figure 3:**
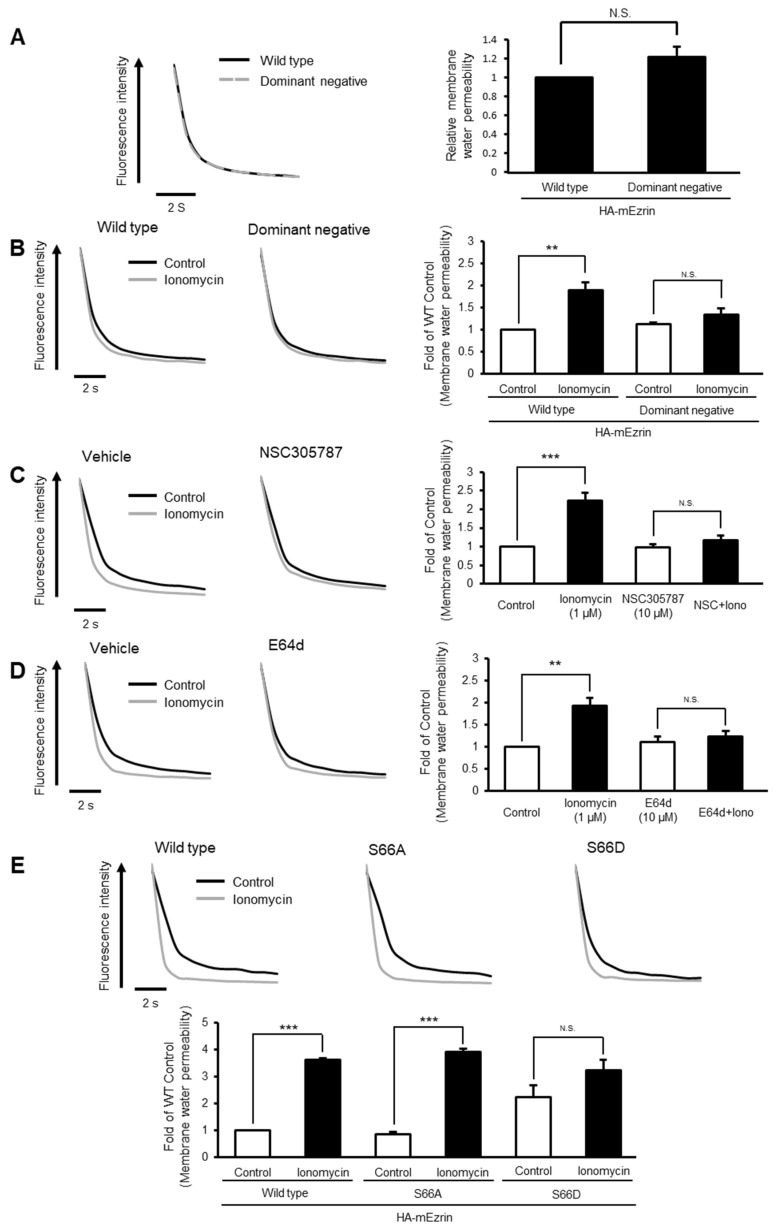
Ezrin inhibition attenuates the ionomycin-induced increase in membrane water permeability in MLE-12 cells. MLE-12 cells were transfected with wildtype or dominant negative (lacking actin-binding domain) ezrin, after which water permeability of plasma membrane was measured by the calcein fluorescence quenching method (**A**)**.** Cells were transfected with wildtype or dominant negative ezrin. The cells were treated with ionomycin (1 µM) for 15 min (**B**) or cotreated with ionomycin (1 µM) and NSC305787 (10 µM) (**C**). Cells were pretreated with E64d (10 µM) for 30 min and then treated with ionomycin (1 µM) for 15 min (**D**). MLE-12 cells were transfected with wildtype, S66A, or S66D (uncleavable mutant) ezrin and then treated with ionomycin (1 µM) for 15 min (**E**). Data represent the mean ± S.E. (*n* = 3–4). ** *p* < 0.01 and *** *p* < 0.001.

Since a basic function of AQP is water transport across the membrane, the AQP-expressed cell has increased membrane water permeability. To examine whether ezrin-mediated membrane trafficking of AQP5 affects this function, we measured membrane water permeability by a calcein fluorescence quenching method. There was no difference in steady-state membrane water permeability between ezrin-WT- and ezrin-DN-transfected MLE-12 cells (Figure 3A). Thus, these data were consistent with the effects on AQP5 translocation. Membrane water permeability did not increase after ionomycin treatment of ezrin-DN- or ezrin-S66D-transfected cells, but it was increased in ezrin-WT or ezrin-S66A-transfected cells (Figure 3B,E). Additionally, NSC305787 or E64d inhibited the increase in membrane water permeability induced by ionomycin (Figure 3C,D). These results suggest that ezrin-mediated stimulation-dependent membrane trafficking of AQP5 is associated with an increase in membrane water permeability.

### 2.4. Cytoskeleton Inhibition Suppresses Stimulation-Dependent Membrane Trafficking of AQP5

**Figure 4:**
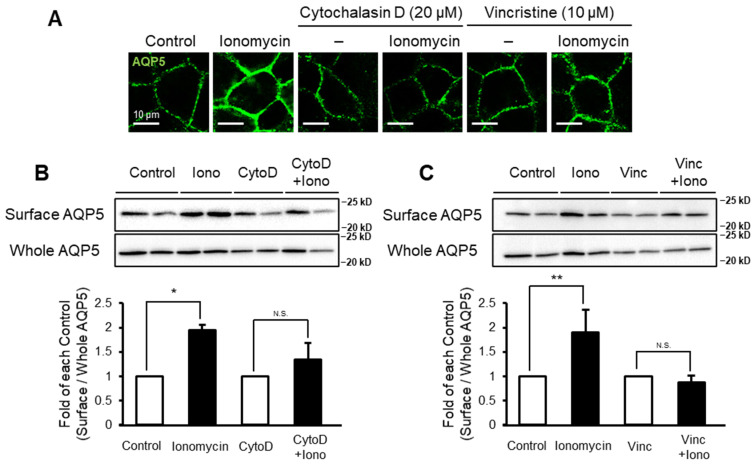
Cytoskeleton is required for ezrin-mediated membrane trafficking of AQP5 in MLE-12 cells. MLE-12 cells were pretreated with cytochalasin D (20 µM) or vincristine (10 µM) for 2 h and then treated with ionomycin (1 µM) for 15 min. Cellular localization of AQP5 (**A**) and the amount of cell surface AQP5 were assessed (**B**,**C**). Data represent the mean ± S.E. (*n* = 3–4). * *p* < 0.05 and ** *p* < 0.01.

Ezrin interacts with the cytoskeleton, which is important for its functions. Therefore, we examined whether the cytoskeleton was involved in the stimulation-dependent membrane trafficking of AQP5. Two hours of cytochalasin D (20 µM) or vincristine (10 µM) pretreatment suppressed AQP5 translocation to the plasma membrane of MLE-12 cells induced by ionomycin (Figure 4A). The increase in the amount of cell surface AQP5 induced by ionomycin was also inhibited by cytochalasin D or vincristine pretreatment (Figure 4B,C). These results suggest that ezrin–cytoskeleton interactions are required for stimulation-dependent membrane trafficking of AQP5.

## 3. Discussion

The major finding of this study is that steady-state plasma membrane localization and Ca^2+^ ionophore-stimulated translocation of AQP5 to the plasma membrane are regulated by different mechanisms and that only Ca^2+^ ionophore-induced translocation of AQP5 requires ezrin. This idea is supported by four lines of evidence. First, the membrane trafficking of AQP5 induced by ionomycin was not observed in ezrin-DN-transfected cells (Figure 1C,D). Second, NSC305787 decreased membrane trafficking of AQP5 induced by ionomycin (Figure 2A,B). Third, E64d suppressed membrane trafficking of AQP5 induced by ionomycin (Figure 2C,D). Finally, membrane trafficking of AQP5 induced by ionomycin was not observed in ezrin-S66D-transfected MLE-12 cells (Figure 2E,F).

ERM proteins consist of ezrin, radixin, and moesin, which bind to membrane proteins. Because the FERM domain is highly conserved among these proteins, it is possible that ezrin-DN inhibited the function of not only ezrin, but also radixin and moesin. NSC305787 inhibited ionomycin-induced AQP5 membrane trafficking in MLE-12 cells at 10 µM (Figure 2A,B). The previously reported IC_50_ values of NSC305787 for ERM proteins are as follows: ezrin, 8.3 µM; moesin, 9.4 µM; radixin, 55 µM [20]. However, among ERM proteins, only ezrin is cleaved by calpain [21]. In this study, calpain inhibition also suppressed stimulation-dependent AQP5 trafficking to the plasma membrane (Figure 2C,D). Moreover, an ionomycin-induced increase in AQP5 trafficking was not observed in MLE-12 cells transfected with ezrin mutant resistant for calpain cleavage (ezrin-S66D), whereas trafficking was observed in the cells transfected with ezrin-WT or ezrin mutant, which is not phosphorylated by PKA (S66A) (Figure 2E,F). Therefore, we excluded the possibility that the inhibitory effect of ezrin-DN and NSC305787 on ionomycin-induced AQP5 plasma membrane trafficking was due to inhibition of radixin and moesin.

The data in this study indicated that ezrin mediated trafficking of AQP5 through its interaction with the cytoskeleton. Cytochalasin D or vincristine, inhibitors of the cytoskeleton, decreased stimulation-dependent membrane trafficking of AQP5 (Figure 4). Consistent with this finding, Tada et al. have reported that interactions between the cytoskeleton and vesicles are important for thapsigargin-induced AQP5 membrane trafficking [23]. 

There are two pathways for plasma membrane trafficking of proteins, such as receptors and ion channels, namely constitutive and stimulation-dependent regulatory trafficking. For example, glucose transporter 4 is translocated to the membrane for glucose uptake by insulin receptor signaling in adipocytes [24,25]. AQP2 is translocalized to the membrane by vasopressin receptor 2 via the cAMP–PKA pathway in the renal collecting duct [26,27,28]. AQP4 is translocalized to the membrane by calmodulin-PKA pathway in astrocyte of rat CNS edema model [29], and trifluoperazine, a calmodulin inhibitor, ameliorates brain edema in acute phase stroke of a photothrombotic mouse model [30]. Moreover, relocalization from storage compartment is involved in regulatory trafficking pathway to the plasma membrane of AQPs [31,32], and cytoskeleton is required to form storage compartment [33]. Our data showed that ezrin-DN did not affect steady-state localization of AQP5 (Figure 1). Taken together, these findings allowed us to conclude that ezrin specifically regulates the regulatory trafficking pathway of plasma membrane localization of AQP5.

Sjögren’s syndrome is an autoimmune disease characterized by malfunction of exocrine glands, which leads to dry eyes and a dry mouth [34,35]. Abnormal distributions of ezrin [36] or AQP5 [37,38] in exocrine gland tissues have been reported in some Sjögren’s syndrome patients. We observed stimulation-dependent regulation of AQP5 by ezrin in not only MLE-12 cells, but also HSG cells (Figure 1E). Therefore, we assume that dysregulation of ezrin-mediated AQP5 trafficking to the plasma membrane may be related to exocrine dysfunctions in Sjögren’s syndrome.

In conclusion, our results provide insights into one aspect of the plasma membrane translocalization mechanism of AQP5. Further elucidation may lead to the development of drugs that modulate the membrane localization of AQPs, as opposed to traditional inhibitors that block the water pore of AQPs [39,40].

## 4. Materials and Methods

### 4.1. Cell Culture

MLE-12 cells were cultured in Dulbecco’s modified Eagle’s medium (DMEM) (Nissui Pharmaceuticals, Tokyo, Japan) supplemented with 5% fetal bovine serum (FBS) (Hyclone^®^, GE Healthcare Life Sciences, Uppsala, Sweden), 1 U/mL penicillin, and 1 µg/mL streptomycin (Gibco, Grand Island, NY, USA). HSG cells were cultured in DMEM supplemented with 10% FBS and penicillin/streptomycin. Doubling time of both cells was 24 h. The cells were split 1:4 (MLE-12) or 1:8 (HSG) during each passage. The passages used for following experiments were 15–35 (MLE-12) or 10–20 (HSG). Cells used for following experiments were cultured until 80% confluent. These cells were not contaminated with mycoplasma.

### 4.2. Antibodies

A rabbit anti-AQP5 antibody (AQP005) was purchased from Alomone Labs (Jerusalem, Israel). A mouse anti-HA antibody (16B12) was purchased from Biolegend (San Diego, CA, USA). A mouse anti-β-actin antibody (A5441) and horseradish peroxidase (HRP)-conjugated rabbit anti-mouse IgG (A9044) were purchased from Sigma-Aldrich (St. Louis, MO, USA). HRP-conjugated donkey anti-rabbit IgG (711-035-152) was purchased from Jackson ImmunoResearch Laboratories (West Grove, PA, USA). Alexa Fluor 488-conjugated goat anti-rabbit IgG (A11008) and Alexa Fluor 647-conjugated anti-mouse IgG (A21235) were purchased from Invitrogen (Carlsbad, CA, USA).

### 4.3. Immunofluorescence

Cells cultured on poly-D-lysine-coated glass slides were fixed with 4% paraformaldehyde (Nacalai Tesque, Shiga, Japan), permeabilized with 0.1% Triton X-100 (MP Biomedicals, Santa Ana, CA, USA) for 30 min, and then blocked with 1% bovine serum albumin (Wako, Tokyo, Japan) for 30 min. The cells were then incubated with the rabbit anti-AQP5 and mouse anti-HA antibodies (1:200 dilution), followed by Alexa Fluor 488-conjugated goat anti-rabbit IgG and Alexa Fluor 647-conjugated goat anti-mouse IgG (1:1000 dilution). The cells were mounted with VECTASHILD mounting medium that contained DAPI (H-1200, Vector Laboratories, Burlingame, CA, USA). Images were obtained under a TCS SP8 confocal microscope (Leica Microsystems, Wetzlar, Germany). Images were taken from at least 3 FOVs in each treatment. The detection sensitivity was adjusted using a sample without primary antibody.

### 4.4. Cell Surface Biotinylation Assay

To evaluate cell surface expression of AQP5, a cell surface biotinylation assay was performed as described previously [41]. Briefly, cell monolayers were washed and then incubated with a cell-impermeable biotin derivative (0.5 mg/mL EZ Link Sulfo-NHS-LC-Biotin, Thermo Fisher Scientific, Waltham, MA, USA) in PBS for 30 min to biotinylate cell surface proteins. Unreacted biotinylated molecules were quenched by addition of 100 mM glycine. Cells were lysed with RIPA buffer (50 mM Tris, pH 7.5, 150 mM NaCl, 1% (*v*/*v*) NP-40, 0.5% (*w*/*v*) deoxycholate, 0.1% (*w*/*v*) SDS, 5 mM EDTA, and 1% (*v*/*v*) proteinase inhibitor cocktail), and immunoprecipitations were performed using streptavidin beads (Thermo Fisher Scientific) to precipitate biotinylated proteins. The beads were washed four times in RIPA buffer as described above, and bound proteins were eluted by incubation in SDS-PAGE sample buffer (10 mM Tris, 3% (*w*/*v*) SDS, 10% (*v*/*v*) glycerol, 0.01% (*w*/*v*) bromophenol blue, and 2.5% 2-mercaptoethanol) for 30 min at 37 °C. Changes in the amount of biotinylated AQP5 mutants were analyzed by immunoblotting.

### 4.5. Immunoblotting

Cell lysates were separated by 12% SDS-polyacrylamide gel electrophoresis and then transferred to a PVDF membrane. The membrane was blocked with 5% non-fat dry milk in PBS with 0.1%-Tween 20 (PBS-T) at room temperature for 1 h and then incubated with the mouse anti-HA antibody (dilution 1:1000) at 4 °C overnight. The membrane was then washed with PBS-T and incubated with horseradish peroxidase-conjugated rabbit anti-mouse IgG (Sigma-Aldrich) at room temperature for 1 h. Immunocomplexes were detected by Super Signal West Pico PLUS Maximum Sensitivity Substrate (Thermo Fisher Scientific). Images were obtained by a ChemiDoc XRS+ System (Bio-Rad Laboratories, Hercules, CA, USA). Band densities were quantified using Image Lab Software (version 6.0, Bio-Rad Laboratories).

### 4.6. Osmotic Water Permeability Assay of the Plasma Membrane

Water permeability across the plasma membrane was measured by a calcein fluorescence quenching method as described previously [42,43]. Briefly, MLE-12 cells transfected with HA-Ezrin-WT or mutants cultured on 96-well clear-bottomed black plates were loaded with 5 µM Calcein-AM (Dojindo, Tokyo, Japan) in DMEM for 90 min. Then, the cells were washed with calcein assay buffer (0.8 mM magnesium sulfate, 5 mM potassium chloride, 1.8 mM calcium chloride, 25 mM HEPES sodium-salt, 183 mM d(-)-mannitol and 5.6 mM d(+)-glucose) twice, and the fluorescence change (λ_ex_ 485 nm, λ_em_ 535 nm) caused by addition of an equal volume of calcein assay buffer containing 100 mM d-(-)-mannitol (for induced Δ50 mOsm hypertonic shock) was measured by a fluorescence plate reader (ARVO X4, Perkin-Elmer, Norwalk, CT, USA).

### 4.7. Statistical Analysis

All data are expressed as the mean ± standard error of the mean. The significance of differences was assessed using one-way ANOVA, followed by the Student–Newman–Keuls test. *p*-values of <0.05 were considered to be significant.
